# The first study on clinicopathological changes in cats with feline infectious peritonitis with and without retrovirus coinfection

**DOI:** 10.14202/vetworld.2023.820-827

**Published:** 2023-04-20

**Authors:** Wassamon Moyadee, Natdaroon Chiteafea, Supansa Tuanthap, Kiattawee Choowongkomon, Sittiruk Roytrakul, Oumaporn Rungsuriyawiboon, Chaiwat Boonkaewwan, Natthasit Tansakul, Amonpun Rattanasrisomporn, Jatuporn Rattanasrisomporn

**Affiliations:** 1 Center for Advanced Studies for Agriculture and Food, Institute for Advanced Studies, Kasetsart University, Bangkok, Thailand; 2 Graduate Program in Animal Health and Biomedical Sciences, Faculty of Veterinary Medicine, Kasetsart University, Bangkok, Thailand; 3 Department of Companion Animal Clinical Sciences, Faculty of Veterinary Medicine, Kasetsart University, Bangkok, Thailand; 4 Faculty of Veterinary Medicine, Rajamangala University of Technology Tawan-ok, Chonburi, Thailand; 5 Department of Biochemistry, Faculty of Science, Kasetsart University, Bangkok, Thailand; 6 Functional Proteomics Technology Laboratory, Functional Ingredients and Food Innovation Research Group, National Center for Genetic Engineering and Biotechnology, National Science and Technology for Development Agency, Pathum Thani, Thailand; 7 Department of Veterinary Technology, Faculty of Veterinary Technology, Kasetsart University, Bangkok, Thailand; 8 Akkhraratchakumari Veterinary College, Walailak University, Nakhon Si Thammarat, Thailand; 9 Department of Pharmacology, Faculty of Veterinary Medicine, Kasetsart University, Bangkok, Thailand; 10 Interdisciplinary of Genetic Engineering and Bioinformatics, Graduate School, Kasetsart University, Bangkok, Thailand

**Keywords:** effusion, feline leukemia virus, feline infectious peritonitis, feline immunodeficiency virus, reverse transcriptase-polymerase chain reaction

## Abstract

**Background and Aim:**

Feline infectious peritonitis (FIP) is an infectious, immune-mediated, and fatal disease in cats caused by a mutant feline coronavirus (FCoV) infection. Feline immunodeficiency virus (FIV) and feline leukemia virus (FeLV) are two common retroviruses that play a role in reducing feline immune function with opportunistic retrovirus infection being a predisposing factor for the development of FIP. This study aimed to evaluate the clinicopathological parameters of FIP in cats with and without retrovirus coinfection.

**Materials and Methods:**

In total, 62 cats presenting with pleural and/or peritoneal effusion at the Kasetsart University Veterinary Teaching Hospital, Bangkok, Thailand, were selected for the study. Effusion samples were collected and a reverse transcriptase-polymerase chain reaction (RT-PCR) assay was performed on all samples using the 3’ untranslated region primer. All FCoV-positive cats were tested for retrovirus infection using a commercial kit (Witness FeLV-FIV [Zoetis]; United States). Clinical signs, hematological, and biochemical parameters of these cats were investigated and grouped.

**Results:**

Of the 62 cats with pleural and/or peritoneal effusion, FCoV was detected in 32, of which 21 were highly suspicious for FIP. The cats suspected of FIP were divided into three subgroups following viral detection. A total of 14 had only FCoV infection (Group A), four had FCoV and FeLV infection (Group B), and three had FCoV, FeLV, and FIV infection (Group C). Of the rest, 11 had definitive diagnoses, which included three being FCoV and FeLV-positive (Group D), and eight were retrovirus-negative (Group E). Mild anemia and lymphopenia were found in cats infected with these three viruses. An albumin-to-globulin ratio lower than 0.5 was found in FIP cats with only FCoV infection.

**Conclusion:**

Typically, cats with clinical effusion and FIP, with and without retrovirus coinfection, had similar hematological findings. Clinical signs, blood parameters, fluid analysis with cytological assessment, and RT-PCR assays could identify better criteria to diagnose FIP with and without retrovirus coinfection.

## Introduction

Feline coronavirus (FCoV) is a positive-sense and single-stranded RNA virus. It is a member of the genus *Alphacoronavirus*, where a mutant of FCoV results in immune-mediated disease in cats. Feline leukemia virus (FeLV) and feline immunodeficiency virus (FIV) are retroviruses that attack cat cells involved in the immune response. All three viruses are among the most common viral diseases found in domestic cats worldwide and affect their quality of life [1–4]. Cats with FCoV are generally healthy, but up to 12% develop feline infectious peritonitis (FIP) [5]. The two clinical forms of FIP are effusive (wet) and granulomatous (dry). Feline infectious peritonitis-related peritoneal or pleural effusions must be distinguished from other causes such as cardiomyopathy, neoplastic, or septic effusions [6]. The most significant initiators of FIP infection in cat care facilities, shelters, and housing are feline retrovirus infections [7]. Infection with FeLV and FIV can cause multiple, variable, and clinical signs depending on the disease stage [3, 8]. The most common outcomes of latent FeLV infection are immunosuppression, bone marrow suppression, and tumorigenesis (lymphoma), whereas FIV can lead to acquired immunodeficiency and an increased risk of opportunistic infections [9–11]. Furthermore, the presence of retroviruses may increase the risk of developing FIP [12].

In general, diagnosing FIP involves using a combination of signalment, clinical signs, blood testing, and specific tests such as a characteristic effusion, coronavirus antibody titers, and virus detection [13]. Typical clinical signs of both types of FIP include anorexia, lethargy, weight loss, and pyrexia, depending on the organ affected [12, 14]. Hematological changes in FIP are usually lymphopenia caused by T-cell apoptosis, neutrophilia, and mild-to-moderate anemia [15–17]. Changes in serum biochemistry in feline FIP are variable and frequently non-specific [12]. The majority of cats with FIP have hyperglobulinemia or hypoalbuminemia, or both, leading to a low albumin-to-globulin (A:G) ratio. Therefore, a high A:G ratio is useful for ruling out FIP [18, 19]. The effusion appearance in cats with FIP is usually straw-colored, thick, clear, turbid, and protein-rich [12, 19]. The total protein of the effusion is usually >3.5 g/dL and often >4.5 g/dL. The total nucleated cell count (TNCC) is often low, ranging from 2,000 to 6,000 cells/µL [20, 21]. Microscopic and cytological examination of FIP effusion reveals it to be pyogranulomatous, a mixture of inflammatory cells on a proteinaceous background [12]. Although these changes are not specific, they can be used to help distinguish a FIP effusion from other effusions such as bacterial pleuritis and neoplasia [22]. A reverse transcriptase-polymerase chain reaction (RT-PCR) assay can be used to detect viral genetic material in tissue or body fluid [23–25]. However, the precise genetic makeup of the FIP virus (FIPV) is not clear [13]. Nonetheless, the highly conserved 3’-untranslated region (3’-UTR) provides additional confirmation of the status of FCoV infection in FIP-suspected cats [23–26]. Cytology and RT-PCR assay of the conserved 3’-UTR have been suggested as the tests of choice for effusions to diagnose FIP [22]. The most studied clinicopathological abnormalities in cats with retroviral infections are similar to those with FIP [21, 27]. However, FIP, FeLV, and FIV are very different from each other. Thus, the specific etiology following high-risk infection in all cats should be determined. The commercial test kit for FeLV and FIV, which has high sensitivity and specificity, utilizes enzyme-linked immunosorbent assay or rapid immunomigration techniques, and it is commonly used clinically to detect FeLV antigen and FIV antibody in whole blood, serum, or plasma [3].

There have been many reports regarding FIP, FeLV, and FIV viruses individually [8, 14, 20], but the differences between individual and coinfection have not been published to the best of our knowledge. This study aimed to investigate changes in the clinicopathological and laboratory parameters of FIP with and without retrovirus coinfection.

## Materials and Methods

### Ethical approval

The study was approved by the Kasetsart University Institutional Animal Care and Use Committee (ACKU62-VET-017), Bangkok, Thailand.

### Study period and location

The study was performed from March 2019 to March 2021 at the Kasetsart University Veterinary Teaching Hospital.

### Animals and experimental design

**Figure-1:**
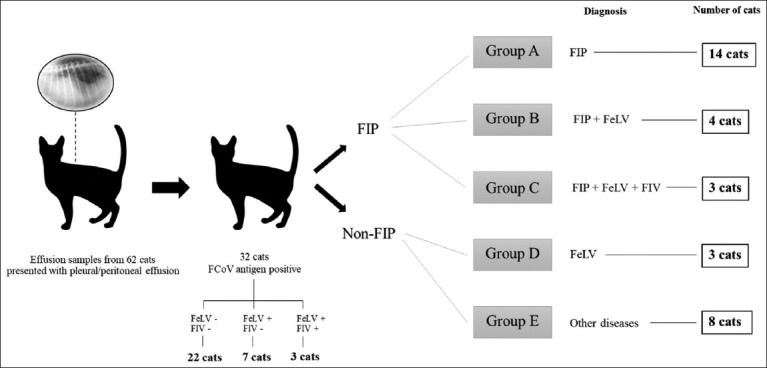
Sample groups in this study.

Cats presenting with fluid accumulation in a body cavity at Kasetsart University Veterinary Teaching Hospital on their initial visit were chosen for analysis. Cats positive for FCoV infection based on RT-PCR assay of their effusion were identified and included in the study. Effusion samples from 62 cats were collected and stored in ethylenediaminetetraacetic acid and plain tubes at −80°C before analysis. Feline coronavirus was detected in 32 samples. Hematological analyses were performed on all cats using an automated cell counter (CELL-DYN 3700; Abbott Laboratories, USA). Serum biochemistry analyses were performed for all cats with the analyses including some or all of the following: Blood urea nitrogen, creatinine (CR), alanine aminotransferase (ALT), total protein, globulin, and albumin using an automated chemical analyzer (Hitachi High-Technologies Co., Japan). Feline leukemia virus and FIV were evaluated using a commercial test kit (Witness® FeLV/FIV, Zoetis, USA). The diagnostic tests for FIP included Rivalta’s test, FCoV antibody test, fluid analysis, cytology, and virus detection based on RT-PCR assay [3, 13, 22]. Cats that died underwent postmortem examination when owner consent was available. Additional diagnostic procedures, based on the differential diagnosis, included radiography, ultrasonography, echocardiography, and histopathology, and they were performed depending on the medical condition. Finally, the cats were divided into the following groups: Only FIP (Group A), FIP and FeLV (Group B), FIP with FeLV and FIV (Group C), FeLV with other diseases (Group D), and other systemic diseases without retrovirus infection (Group E). The groupings were based on the medical records and definitive diagnosis of the 32 cats (Figure-1).

### Sample preparation, RNA extraction, and viral nucleic acid detection

Effusion samples were prepared as 10% (w/v) suspensions with sterile phosphate-buffered saline and centrifuged for 10 min at 1000× *g* with only the supernatant used for further assay. Total RNA was extracted using a viral RNA purification kit according to the manufacturer’s instructions (EZNA Viral RNA Kit, Omega Bio-Tek, GA, USA). Subsequently, cDNA was retrieved using a RevertAid First Strand cDNA Synthesis Kit (Thermo Scientific Inc., MA, USA). All samples were tested for the highly conserved 3’-UTR of Type I and Type II FCoV (sense P205: 5’-GGCAACCCGATGTTTAAAACTGG-3’; antisense P211: 5’-CACTAGATCCAGACGTTAGCTC-3’; targeting a 223 bp) using Phusion Hot Start II High-Fidelity DNA Polymerase (Thermo Scientific Inc.) [23].

### Statistical analysis

Descriptive analyses of signalment and abnormal increases or decreases in blood parameters were summarized as percentages.

## Results

### Animals

Of the 62 effusion samples, FCoV was detected in 32, and 10 had detectable FCoV with retrovirus coinfection, seven had FeLV, and three had FeLV and FIV. The inclusion criteria for FIP diagnosis were based on the general guidelines [12, 13, 19]. All 32 cats had a high suspicion for being infected with FIP. The 32 cats included 14 (No. 1–14) infected with only FCoV, 4 (No. 15–18) infected with FCoV and FeLV, 3 (No. 19–21) infected with FCoV, FeLV, and FIV, 3 (No. 22–24) infected with FCoV and FeLV which clinically had FeLV, and eight with other systemic diseases or incomplete diagnostic results. These cats were divided into five groups: A, B, C, D, and E, respectively (Table-1). Of the three cats in Group D, No. 22 and No. 24 had mediastinal lymphoma, and No. 23 had pancreatitis with a septic peritoneal effusion identified as *Escherichia coli* and *Staphylococcus* spp. Among the eight cats in Group E, No. 25 and No. 31 had no cytological results. No. 25 had a red-brown effusion, and ultrasonography identified right-sided renomegaly with a tentative diagnosis suggestive of FIP. No. 26 was lost to follow-up and had no final diagnosis. No. 27 was diagnosed with a hepatic tumor and a polycystic kidney. Cytological examination of the effusion found neoplastic cells and a possible bile duct carcinoma or pancreatic carcinoma. No. 28 had chronic heart failure with unclassified cardiomyopathy (UCM) confirmed by echocardiography. No. 29 had a serosanguineous fluid appearance, and the final diagnosis was peritoneopericardial diaphragmatic hernia with a hepatic cyst. No. 31 was diagnosed with chronic kidney disease (CKD) and hypertrophic cardiomyopathy confirmed by echocardiography. No. 30 had CKD and chylothorax, and No. 32 had CKD and pyothorax with *Pasteurella multocida* cultured from the pleural effusion.

**Table-1:** List of 32 cats in the study, including signalments, retrovirus status, clinical effusion sites, cytological evaluation of effusions, and serum albumin-to-globulin (A:G) ratio.

No.	Age (year)	Gender	Breed	Fluid analysis		FeLV and FIV status	Serum A:G ratio

				Effusion site	Cytological diagnosis		
1	2	Male	DSH	Pleural	Non-septic exudate	-/-	0.43
2	0.7	Female	DSH	Peritoneal	Non-septic exudate	-/-	0.39
3	0.8	Male	Scottish fold	Pleural	Modified transudate	-/-	0.29
4	0.4	Female	British shorthair	Peritoneal	Modified transudate	-/-	0.35
5	0.5	Male	DSH	Peritoneal	Modified transudate	-/-	0.54
6	1.1	Male	DSH	Pleural	Non-septic exudate	-/-	0.45
7	1.7	Male	Scottish fold	Peritoneal	Non-septic exudate	-/-	0.76
8	0.7	Female	DSH	Peritoneal	Non-septic exudate	-/-	0.67
9	0.4	Male	DSH	Peritoneal	Modified transudate	-/-	0.38
10	0.9	Female	DSH	Peritoneal	Modified transudate	-/-	0.46
11	0.5	Male	Persian	Pleural	Non-septic exudate	-/-	0.35
12	0.5	Male	DSH	Peritoneal	Non-septic exudate	-/-	0.35
13	1.7	Male	DSH	Peritoneal	Non-septic exudate	-/-	0.42
14	1	Female	DSH	Peritoneal	Modified transudate	-/-	0.32
15	1	Male	Persian	Peritoneal	Non-septic exudate	+/-	0.72
16	3.2	Male	DSH	Pleural	Non-septic exudate	+/-	0.43
17	3	Male	DSH	Pleural	Non-septic exudate	+/-	0.51
18	0.7	Female	DSH	Pleural	Non-septic exudate	+/-	0.42
19	1.3	Male	Persian	Peritoneal	Modified exudate	+/+	0.68
20	0.9	Female	DSH	Peritoneal	Non-septic exudate	+/+	0.7
21	3.6	Female	DSH	Pleural	Non-septic exudate	+/+	0.64
22	2	Male	DSH	Pleural	Chylous effusion	+/-	0.78
23	4	Female	DSH	Peritoneal	Modified transudate	+/-	0.6
24	2	Male	DSH	Pleural	Chylous effusion	+/-	0.7
25	2.1	Male	Abyssinian	Peritoneal	N/A	-/-	0.2
26	17	Female	DSH	Pleural	Modified transudate	-/-	N/A
27	12.3	Male	Persian	Peritoneal	Neoplastic effusion	-/-	0.8
28	10	Male	DSH	Peritoneal and pleural	Transudate	-/-	1.0
29	3	Male	Persian	Pleural	Modified transudate	-/-	1.1
30	13.2	Female	DSH	Pleural	Chylous effusion	-/-	0.5
31	14.7	Male	DSH	Pleural	N/A	-/-	0.4
32	18	Female	DSH	Pleural	Pyothorax	-/-	0.61

No. 1–14=Cats in Group A, No. 15–18=Cats in Group B, No. 19–21=Cats in Group C; No. 22–24=Cats in Group D, No. 25–32=Cats in Group E. +=positive, –=negative. N/A=Not available, DSH=Domestic shorthair

The median ages for Groups A, B, C, D, and E were 8 months, 2 years, 1.3 years, 2 years, and 14.8 years, respectively. Of the 14 cats in Group A, 10 (71.43%) were <1 year of age, and the remaining 4 (28.57%) were 1–1.6 years of age. Retroviruses were detected in 10/10 (100%) cats under 4 years of age. Of the cats in Group E, 2 (25%) were 2–3 years of age, while the others (75%) were 10–18 years of age. Male cats were more likely to be infected with FCoV (62.5%, 20/32), and 6/10 (60%) FeLV-positive cats were male. In addition, 9/32 cats (28.12%) were purebred, consisting of Persian (5), Scottish fold (2), British shorthair (1), and Abyssinian (1). The other 23/32 (71.88%) were domestic shorthair cats. Of the 32 cats, 16 had peritoneal effusion, 15 had pleural effusion, and one had both peritoneal and pleural effusions.

### Fluid analysis and cytology

Fluid analysis and cytological examination of the 32 samples showed non-septic exudate (13), modified transudate (10), chylous effusion (3), pyothorax (1), transudate (1), neoplastic effusion (1), and one with no result. Of the 14 cats in Group A, the effusion protein levels were 4.2–7.3 g/dL (median 6 g/dL). The TNCC was 460–25,300 cells/μL (median 4095 cells/μL) with a mixture of non-degenerate neutrophils and macrophages with some lymphocytes, consistent with modified transudates, or non-septic exudates. For the four cats in Group B, protein levels were 3.6–7.7 g/dL (median 4.9 g/dL), and the TNCC was 633–16,700 cells/μL (median 5,365 cells/μL). The three cats in Group C had effusion protein levels of 4.4–5.5 g/dL (median 5 g/dL), and the TNCC was 12,740–46,250 cells/μL (median 37,336 cells/μL). Two of three cats in Group D had TNCC values of 633 and 16,700 cells/μL, protein levels in the effusions of 3.9 and 4 g/dL, and lymphoblasts, while the other cat in the group had no TNCC or effusion protein recorded. The protein levels in the effusions of the six cats in Group E were 2.2–5 g/dL (median 2.5 g/dL). Five of eight cats had a TNCC of 210–3233 cells/uL (median 1590 cells/μL), one cat with a pyothorax had 274,612 cells/uL, and the others in the group did not have results recorded.

### Hematological findings and serum biochemical changes

As shown in Table-2, the packed cell volume (PCV) decreased in all groups except for Group D. The median red blood cell and hemoglobin values were low in Group B. Of the cats in Group A, 57% had mild anemia (PCV 20%–29%), and 14% had moderate anemia (PCV 15%–19%). The seven cats in Groups B and C all had moderate anemia. The median white blood cell value was normal in all groups. All cats in Groups A and B had low levels of albumin, while all cats in Groups C and D were normal. The median globulin level in all groups was normal. Five cats in Group A and one cat in Group B had hyperglobulinemia. The median A:G ratios for groups A, B, C, D, and E were 0.4, 0.47, 0.68, 0.78, and 0.62, respectively. Blood urea nitrogen, CR, and ALT levels for almost all cats were normal. Two cats (No. 4 and No. 5) in Group A and one cat (No. 21) in Group C had increased ALT levels more than 3 times normal. None of the cats with FIP in Groups A, B, or C had azotemia.

**Table-2:** Hematological and biochemical parameters of feline coronavirus positive cats between groups.

Measure	Unit	Normal Range	Group A (n = 14)			Group B (n = 4)			Group C (n = 3)			Group D (n = 3)			Group E (n = 8)		
				
			Mean	Median	Range	Mean	Median	Range	Mean	Median	Range	Mean	Median	Range	Mean	Median	Range
Hematology																	
PCV	%	30–45	27.86	28.05	18.1–44.3	27.58	27.55	20.7–34.5	28.87	28.30	22.6–35.7	33.13	32.40	28.1–38.9	23.15	24.65	15–27.5
RBC	×10^3^/uL	5–10	6.62	6.38	3.81–10.4	5.46	5.51	3.74–7.06	6.08	6.39	3.76–8.08	6.64	6.75	5.61–7.56	5.79	5.64	3.18–8.54
HGB	mg%	10–15	9.23	9.11	5.65–14.2	8.42	8.48	6.81–9.93	10.50	10.8	8.2–12.5	10.40	10.2	8.39–12.6	7.77	8.35	4.8–10.1
WBC	×10^3^/uL	5.5–19.5	14.86	13.6	7.14–37.1	16.22	16.3	11.2–21.1	10.44	10.46	7.36–13.5	20.73	16.60	12.1–33.5	18.02	18.38	5.51–29.24
NEU		2.5–12.5	10.86	10.82	6.43–19.32	14.31	14.68	10.98–16.88	7.24	8.5	3.39–9.83	18.35	15.6	10.65–28.81	15.51	15.81	4.52–24.85
LYMPH		1.5–7.0	1.62	1.2	0.57–5.37	1.23	1.18	0.22–2.32	2.56	3.09	0.42–4.19	1.68	0.36	0.33–4.36	1.68	1.4	0.50–4.09
MONO		0–0.9	0.53	0.28	0–1.59	0.53	0.42	0–1.27	0.41	0.23	0.21–0.88	0.50	0.33	0–0.36	0.34	0.4	0–0.5
EOS		0–0.8	0.12	0	0–0.68	0.16	0	0–0.63	0.14	0	0–0.4	0.46	0.34	0.33–0.73	0.31	0	0–0.1
PLT	×10^3^/uL	200–800	280	269	168–460	306	312	200–400	224	224	203–245	267	300	200–300	351	325	200–550
Blood chemistry																	
BUN	mg%	19–34	17.25	16	12–23	16.25	16	12–22	25.33	22	17–37	31.33	22	17–55	44.86	33	14–100
Creatinine	mg/dL	0.9–2.2	0.99	0.95	0.55–1.48	0.94	0.94	0.63–1.23	1.16	1.13	0.87–1.47	1.34	1.31	0.96–1.76	2.25	1.62	0.82–5.63
ALT	IU/L	25–97	92	32	15–475	45	53	17–58	235	70	16–619	29	31	19–36	172	58	31–764
Total protein	mg/dL	5.8–7.8	7.20	7.3	4.3–9.4	6.68	6.8	5–8.1	7.30	7.3	6.4–8.2	6.70	6.6	6.6–6.9	7.49	6.8	5.1–12
Albumin	mg%	2.6–4.2	2.06	2.1	1.5–2.4	2.20	2.2	2–2.4	2.93	3	2.6–3.2	2.77	2.8	2.6–2.9	2.53	2.6	1.9–3.1
Globulin	mg%	2.6–5.1	5.14	5.25	2.8–7.3	4.48	4.65	2.9–5.7	4.37	4.3	3.8–5	3.93	3.8	3.7–4.3	4.96	4.2	2.5–10.1
A: G ratio			0.42	0.4	0.29–0.67	0.52	0.47	0.42–0.72	0.67	0.68	0.64–0.7	0.77	0.78	0.74–0.78	0.65	0.62	019–1.15

ALT=Alanine aminotransferase; A:G ratio=albumin-to-globulin ratio, BUN=Blood urea nitrogen, EOS=Eosinophils, HGB=Hemoglobin, LYMPH=Lymphocytes, MONO=Monocytes, NEU=Neutrophils, PCV=Packed cell volume, PLT=Platelets, RBC=Red blood cell, WBC=White blood cell

## Discussion

A definitive diagnosis of FIP is difficult due to the similarities in clinical signs between FIP and other feline diseases [23]. Nevertheless, a cat presenting with hyperproteinemia due to hyperglobulinemia, fever, and high protein ascites and/or pleural effusion may be highly suggestive of FIP [21, 28]. Reverse transcriptase-polymerase chain reaction assays are available, but the test is limited in its ability to differentiate between feline enteric coronavirus and FIPV [12, 29]. Feline coronavirus RNA can be detected in tissue, effusion, blood, cerebrospinal fluid, and aqueous humor samples from cats with suspected FIP [30]. Effusion is the recommended sample for RT-PCR testing because it often contains FCoV RNA, and the presence of viral RNA in fluid is one of the most reliable diagnostic indicators of FIP [31]. Furthermore, the effusion in suspected cases of FIP is extremely helpful for diagnostic purposes such as fluid analysis, cytology, and immunostaining [12, 13, 19, 32]. Young cats were the most commonly affected age group in a worldwide survey of FIP [17, 32–34]. The cat’s age at the time of infection is likely the most relevant host factor affecting the clinical outcome of FeLV infection [35]. Infections with both FeLV and FIV exhibit chronic characteristics that develop through different disease processes [36]. In the present study, cats with FIP alone were younger than those with retrovirus coinfection. However, FIP has also been reported in middle-aged and senior cats [37]. A study in Australia found cats with FIP to be 2 months–15 years of age. In the present study, one senior cat (No. 31) in Group E was excluded because of the lack of cytological results, but the clinical signs suggested FIP. Depending on the viruses induced, FeLV might be related to a FCoV mutation or immunocompromise, resulting in the development of FIP. In future studies, the relationship between these two groups should be analyzed. The most observed purebred cats in the study were Persians and Scottish folds as has been recently reported [14]. Based on a worldwide survey, male cats are more susceptible to infection with retrovirus and more likely to develop FIP [12, 38]. In the present study, the number of male cats with FIP and retrovirus coinfection was higher than that for females.

Hematological and serum biochemical findings reported in association with FIP are non-specific. The common hematological changes include anemia, neutrophilia, and lymphopenia [12]. Lymphopenia is most frequently observed in cats with FIP and FeLV infection, whereas FIV has a low prevalence [25]. Secondary immune-mediated vasculitis in FIP with effusion results in hypoalbuminemia, which may be a cause of protein loss [33]. In the present study, the albumin level tended to be very low in cats with FIP alone and cats with both FIP and FeLV, while cats with FIP, FeLV, and FIV had normal levels. Hypoalbuminemia in cats can be found in various conditions such as FIP, tumors, and chronic diseases; however, it is possible to have normal or low albumin levels in cats with FIP. There was a high frequency of cats with hypoalbuminemia (85.71%) in Group A (FIP), which is similar to a previous report by Moyadee *et al*. [14]. Hyperproteinemia has been documented in cats with FIP, but more commonly in the non-effusion form. In the present study, the low percentage of hyperproteinemic cats might have been caused by protein loss, not only of albumin, into body cavities, whereas the median total protein levels in all groups were in the normal reference range. Globulin levels are usually elevated in cases of FIP, but in the present study, only three of 14 cats with FIP had hyperglobulinemia compared with the reference interval. The A:G ratio has been reported to be a good diagnostic tool for FIP with an A:G ratio >0.8 helping to rule out FIP [28, 39], while an A:G ratio of 0.4–0.5 is strongly suggestive of FIP [13, 14]. However, Jeffery *et al*. [18] reported that the A:G ratio had a high-risk of being false-positive for FIP due to low positive predictive values of A:G <0.8 and <0.6 at 12.5% and 25%, respectively. Although blood biochemical changes in all three viral infections are not specific, they can be used to predict the progression of infection or to monitor the response during treatment. One limitation of the present study was the duration of treatment and infection, and this needs to be clarified and investigated in future studies.

From cytological findings, FIP effusion typically consists of neutrophils, macrophages, and fewer lymphocytes [12]. Cell types can be helpful in the differential diagnosis of suspected FIP with retrovirus infection. However, a confirmatory test and immunohistochemistry should be performed to identify the cases as single or complex diseases. Chyle in the thoracic cavity may be the result of multiple diseases, such as congestive heart failure, tumors, and idiopathic chylothorax [21, 28]. Although the characteristic effusion in FIP is a straw-yellow color and high in protein, it has been reported that chylous effusion is associated with vasculitis, such as FIP [40, 41]. In the present study, chylous effusion was found in FeLV cats No. 19 and No. 21 and was related to mediastinal lymphoma. The histological findings of vasculitis, and the appearance of FCoV particles in macrophages or monocytes, may be helpful in confirming the diagnosis of lymphoma concomitant with FIP.

In the present study, three cats in Group E (No. 27–29) had A:G levels >0.8, the cutoff level. Cat No. 29, a UCM cat, had effusion samples collected at 4 different time points during treatment with FCoV being positive in all samples. However, the cat did not die immediately and is still alive to date. Another report suggested that non-immune organs, such as the heart and liver, might be affected by systemic inflammatory diseases such as FIP [42]. Therefore, the results of the present study strongly suggest that a high A:G ratio is useful to rule out FIP.

Demonstration of FCoV RNA in the ascites of cats with suspected FIP using an RT-PCR assay was a useful indicator for diagnosis. However, the results of the RT-PCR assay for diagnosing FIP should be interpreted in conjunction with clinical signs, because it is possible that the cat may have more than one disease leading to an effusion.

## Conclusion

The present study is the first, to the best of our knowledge, that reports clinical and laboratory findings for FIP with and without retrovirus coinfection. The results showed that clinical FIP with and without retrovirus infection did not differ in their hematological parameters. An A:G ratio <0.5 is primarily found in cats with only FCoV infection. Importantly, one cat may have multiple infections or multiple diseases simultaneously. A limitation of this study was the duration of treatment and the point of infection which needs further clarification and investigation. In future studies, the relationship between these two groups should be analyzed. Consequently, all sick cats should be screened for retrovirus infection, and the diagnosis of suspected FIP should be carried out carefully. Future studies will require a larger sample size and correlation analysis to increase the discrimi­nating power of parameters for surveying the difference between FIP with and without retrovirus coinfection.

## Data Availability

The supplementary data can be obtained from the corresponding author upon a request.

## Authors’ Contributions

JR: Conceived and supervised the study and manuscript editing. WM: Conducted the study, sample collection, interpreted the results, and drafted the manuscript. NC, ST, and AR: Sample collection and laboratory work. KC, SR, OR, CB, and NT: Research coordination and reviewed and revised the manuscript. All authors have read, reviewed, and approved the final manuscript.
